# Facets of optically and magnetically induced heating in ferromagnetically doped-NaYF_4_ particles

**DOI:** 10.1088/2399-6528/acde43

**Published:** 2022-06-22

**Authors:** Snigdhadev Chakraborty, Gokul Nalupurackal, M Gunaseelan, Srestha Roy, Muruga Lokesh, Jayesh Goswami, Priyankan Datta, Pallab Sinha Mahapatra, Basudev Roy

**Affiliations:** 1 Department of Physics, Quantum Centres in Diamond and Emergent Materials (QuCenDiEM)-group, Micro Nano and Bio-Fluidics (MNBF)-Group, Indian Institute of Technology Madras, Chennai, 600036, India; 2 Department of Mechanical engineering, Indian Institute of Technology Madras, India; 3 Department of Physics, Rathinam Research Hub, Rathinam College of Arts and Science, Coimbatore, 641021, India

**Keywords:** upconversion particle, ferromagnetic, photothermal therapy, magneto thermal therapy

## Abstract

Upconverting particles like Yb and Er-doped NaYF_4_ are known to heat up after illumination with light at pump wavelength due to inefficient upconversion processes. Here we show that NaYF_4_ particles which have been co-doped not only with Yb and Er but also Fe improves the photothermal conversion efficiency. In addition, we show for the first time that alternating magnetic fields also heat up the ferromagnetic particles. Thereafter we show that a combination of optical and magnetic stimuli significantly increases the heat generated by the particles.

## 1 Introduction

Cancer therapy has advanced significantly over the last few decades but yet cancer remains one of the most deadly diseases in the world. The conventional methods of treatment such as radiation therapy, microwave ablation, magnetic-hyperthermia, or chemotherapy have contributed significantly to this area [1–5]. Some of these methods rely on macroscopic heat sources to kill the diseased tissue. Photothermal therapy using nano/microparticles has been introduced as yet another technique to treat patients [6–10]. In this regard, photothermal therapy by employing upconverting particles (UCPs) has gained recent research interest since these particles themselves emerge as multi-modal probes to their environment [11, 12].

Upconverting particles are known for photon-energy conversion via multiphoton processes from NIR to the visible range with tunable efficiencies [13–15]. UCPs have extensively been used for *invivo* imaging [16, 17] and photothermal therapy simultaneously [18, 19]. Since the pristine upconverting particles such as NaYF_4_: Er^3+^, Yb^3+^ (NYF) have been reported with poor upconversion efficiency, with the remainder of the energy converted to heat, the core–shell configuration of UCPs with metals/dielectric have been investigated [11, 20] to improve the non-radiative transfer and, consequently, the heating efficiency. Due to the enormous heat produced by these core–shell particles, it is very hard to optically trap them for the purpose of micromanipulation.

In this work, we report application of NaYF_4_: Er^3+^, Yb^3+^ based UCPs which are codoped with different concentrations of iron (Fe) towards photothermal and magnetothermal heating. These particles also have adequate refractive index values that enable optical trapping [21]. Earlier studies have shown the enhancement in emission as well as lattice heating of NaYF_4_, Er^3+^, Yb^3+^ UCPs with Fe-doping [22, 23]. We show that these have better efficiency at heat generation upon illumination with 980 nm Laser light than those without iron doping. At the same time, the presence of iron in the lattice makes the particles ferromagnetic with saturation magnetization values as high as 3 Am^−1^ [21]. We first show the heating effects of these iron-codoped UCPs (Fe-15 NYF and Fe-30 NYF) through multi-cycle hysteresis losses, at an applied field frequency of 50 Hz. The conventional magnetic hyperthermia technique employs alternating magnetic fields with frequencies in the order of hundreds of kilo-Hertz [24, 25]. The precise control over the translations[26–28] and rotations [29–31], of NYF-based upconverting particles by optical and magnetic means is an added advantage of these particles when it comes to micromanipulation and remote actuation requirements.

## 2 Materials and methods

### 2.1 Preparation of Fe- doped upconverting particles

A basic hydrothermal method [32] is followed to prepare the Fe-doped NaYF_4_:Yb,Er upconverting particles [21, 28]. To prepare 15 at% Fe doped NaYF_4_:Yb,Er (Fe-15 NYF), 0.126M yttrium nitrate (Y(NO_3_)_3_,63 at% of Y) and 0.3M of sodium citrate (Na_3_C_6_H_5_O_7_) were mixed with 14 ml of water by vigorous stirring for 10 min. After this, 40m Mytterbium nitrate (Yb(NO_3_)_3_, 20 at% of Yb),4 mM erbium nitrate(Er(NO_3_)_3_, 2 at% of Er) and 30 mM ferric nitrate(Fe(NO_3_)_3_,15 at% Fe) in 21 ml of deionized water were added in previously made solution, resulting in a white solution. Then, 0.5 M of Sodium Fluoride (NaF) in 67 ml of water was added to the solution. The solution turns from white to transparent and is stirred for 1 h. The transparent solution was then transferred to a tightly sealed Teflon-lined autoclave (200 ml). Then, this is heated in a muffle furnace at 200 °C for 12 h. After cooling naturally to room temperature, the solution was washed with water and ethanol four times. After drying at 100 °C for 12 h, the powdery sample was obtained. NaYF_4_:Yb, Er, 30 at% (Fe-30 NYF) particles were also prepared by similar steps except for modification in the concentration of 0.06M of ferric nitrate (Fe(NO_3_)_3_,30 at% Fe) and 0.096 of ytterbium nitrate (Yb(NO_3_)_3_, 48 at% of Yb). The physical and chemical properties of the particles are reported in our previous work [21].

### 2.2 Optical tweezers setup

**Figure 1:**
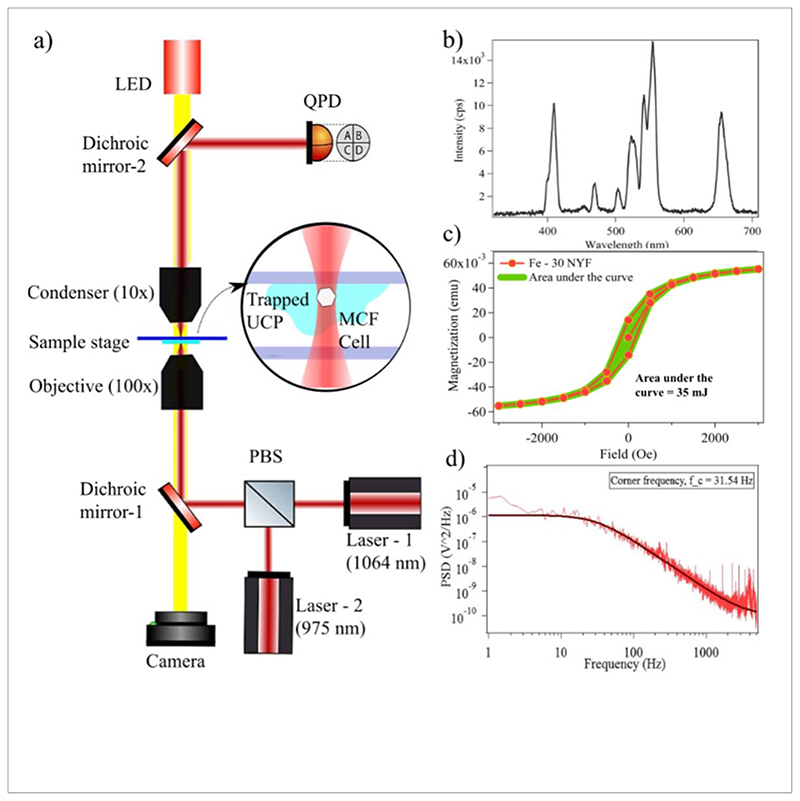
(a) Shows the schematic diagram of the experiment. A Fe-15 NYF is trapped inside the cell using both 975 nm and 1064nm lasesa to determine the particle’s photothermal properties. (b) The emission spectra of the particle are shown. In (c), the room temperature vibrational magnetometric data of Fe-30 NYF is shown to ascertain its ferromagnetic properties. The magnetization of the particle saturates at a value, 3A m.^−1^ A Fe-15 NYF is trapped in a water medium with 1064 nm Laser and the corresponding power spectral density, which follows a Lorentzian is also shown in (d).

We show the experimental setup in figure 1 (a). This setup comprises an OTKB/Moptical tweezers kit from Thorlabs, USA. We use a 100x, 1.3 numerical aperture (N.A) oil immersion type objective from Olympus, E plan 10x with 0.25 N.A. An (air immersion) condenser from Nikon, a diode Laser of wavelength 1064 nm (Lasever, China), and a butterfly Laser of wavelength 975 nm (Thorlabs, USA). The sample chamber is formed using upconverting particles suspended in 20 *μ*l of deionized water or cells with buffer, placed between a coverslip (Blue star, number 1 size, English glass) and glass slide (Labtech,26mm × 76mm × 1.1mm) and clipped to the sample stage. The sample stage is illuminated by a white light LED through dichroic mirror-2 and a CMOS camera is placed to image the sample plane. Two dichroic mirrors are placed to reflect the Laser and transmit the visible light toward the camera. To ascertain, the thermal characteristics in bulk, the standard cuvette-Laser-thermister arrangement is employed [33].

### 2.3 Preparation of *MCF –* 7 cells

MCF-7 (Human Breast Cancer cells, National Center for Cell Science, Pune, India) used for our experiment were prepared according to standard protocols as described in [34]. The cells were grown on gelatin-coated glass coverslips and infused with upconversion particles. The glass coverslips were precleaned with detergent followed by an acid wash with conc. HNO_3_ and UV treatment for one hour for complete sterilization. A thin coating of 0.1% gelatin was applied on these coverslips and they were incubated at 37 °C for about one hour. Before seeding the cells, the coverslips are washed with 1X PBS. The cells are suspended in Dulbecco’s Modified Eagle’s Media (DMEM) with 10% Fetal Bovine Serum (Gibco) and 1% penicillin-streptomycin (Gibco) to protect the cell suspension from bacterial contamination. From the cell suspension containing about 10^5^ cells mL^−1^, 20 *μ*l was placed at the center of the coverslip and incubated at 37 °C with 5% CO_2_ flow for one hour to allow the cells to attach to the gelatin-coated glass surface. Once they are well attached, fresh media containing a diluted suspension of upconversion particles are added to it and incubated for 18–20 h to provide sufficient time for the cells to ingest the upconversion particles via endocytosis.

## 3 Theory

### 3.1 Determination of photothermal and magnetothermal conversion efficiencies

The calculations of the heating efficiency of 975 nm Laser on different upconversion-based particles are already reported [24, 35, 36]. By the law of conservation of energy,

(1) $$\sum_{i=1}^{\infty }m_{i}c_{i}\frac{dT}{dt}=Q_{ucp}+Q_{b}-Q_{s}$$

Where, *m_i_* and *c_i_* are the mass and specific heat capacity of the water where the sample is dispersed (the temperature of which is measured by the thermometer), *Q_ucp_* is the heat produced due to absorption of 980 nm radiation by UCP per second, *Q_b_* is the heat-induced per second by the water independently (baseline), and *Q_s_* is the heat transfer away from the surface by air [36]. Here,

(2) $$Q_{ucp}=\text{I}\eta (1-10^{-A_{980}})$$

And by the law of heat convection,

(3) $$Q_{s}=hS(T-T_{amb})$$

Where, *I* is the incident Laser power, *η* is the heat conversion efficiency for 980 nm Laser, *A*_980_ is the absorbance of upconversion particles at 980 nm, *h* is the heat transfer coefficient, *S* is the surface area of the cuvette in which sample is kept, *T_amb_* is the ambient temperature and *η* is the thermal conversion efficiency. *Q_b_* is found to be 26.2 mW in our case (the energy absorbed by the water). To find *hS*, driving force temperature (*θ*) and a time constant cooling (*τ*) are introduced.

(4) $$\theta (T)=\frac{T-T_{amb}}{T_{\max}-T_{amb}}$$

(5) $$\tau =\frac{\sum_{n=1}^{\infty }m_{i}c_{i}}{hS}$$

At *T* = *T*_max_, $\frac{dT}{dt}=0,$ implies *Q_ucp_* + *Q_b_* – *Q_s_* = 0, then *Q_ucp_* + *Q_b_* = *hS* (*T*_max_ – *T_amb_*). The Laser is turned off once the temperature rise caused by photothermal conversion reaches saturation (T_max_). There is no new heat added to the particle and surroundings. Heat is only redistributed between the particle and the surrounding water, such that (*Q_UCP_* + *Q_b_*) is zero during this period. However, the heat lost by the sample via heat transfer away from the surface by air persists (*Q_s_*) as it begins to cool till the temperature reaches *T_amb_*. From equation (2) and 3,

(6) $$\eta =\frac{hS(T_{\max}-T_{amb})-Q_{b}}{I(1-10^{-A_{980}})}$$

Also, by substituting equation (5) in (3) at T = T_max_, the rate of change in driving force temperature is given by,

(7) $$\frac{d\theta }{dt}=\frac{1}{\tau }(\frac{Q_{ucp}+Q_{b}}{hS(T_{\max}-T_{amb})}-\theta )$$

During the cooling period, Q_*ucp*_ + Q_*b*_=0. Hence equation (7) is reduced to,

(8) t=−τlnθ

Also, to find the thermal conversion efficiency of Fe-30 NYF under an alternating magnetic field of peak-to-peak amplitude 300 G and frequency 50 Hz, 100 g L^−1^ of the sample is kept exposed to the field. We observe that there is no change in the temperature of pure water under exposure to the same field. By applying the law of conservation of energy again,

(9) $$\sum_{i=1}^{\infty }m_{i}c_{i}\frac{dT}{dt}=Q_{mag\;}-Q_{s}$$

Here, *Q_mag_* represents the heat generated by 30% Fe doped UCP in water (100 mg in 1 ml of water) due to the hysteresis losses, per second. It may be noted that the magnetization of 30% Fe-doped UCP saturates at 300 G dc field. Hence, the applied field is sufficient to complete one full cycle of hysteresis for the particles.

Further, just as in the previous case, at the maximum temperature, LHS of equation (9) becomes zero. ⇒Q_*mag*_ = Q_*s*_. Where Q_*s*_ is given by equation (3). We define the net energy lost by the particles per second per hysteresis loop as Q_*h*_ under ideal conditions and Q_*mag*_ be the corresponding heat measured experimentally per second per hysteresis. Then,

(10) $$Q_{mag\;}=\gamma Q_{h}$$

Here, *γ* is defined as the magneto-thermal conversion efficiency. Once the magnetic field is turned off, the temperature-fall of the sample follows the same law. Hence, the time constant (*τ*) for cooling is defined by equation (5). And the linear fit between t and -ln *θ* (figure 3(d), 300 G curve) yields the value of *τ* as 249.36s. The thermal conversion efficiency(*γ*) in this case is reduced to,

(11) $$\gamma =\frac{hS(T_{\max}-T_{amb})}{Q_{h}}$$

### 3.2 Determination of magneto-hyperthermic parameters

One of the important steps when characterizing a magnetic microparticle as a heating agent is to determine its specific loss power (SLP) and intrinsic loss power (ILP). SLP is defined as the electromagnetic power lost per magnetic material mass unit and is expressed in watts per kilogram[37]. It is given by,

(12) $$SLP=\frac{C_{v}}{X_{Fe-NYF}}\times \frac{dT}{dt}$$

Where, *C_v_*, *X_Fe-NYF_* are the specific heat capacity of the medium in which the particles are immersed and the mass fraction of Fe-NYF particles in the medium respectively.

ILP is represented as the SLP normalized by the frequency and the square of the field intensity [38].

(13) $$ILP=\frac{SLP}{f\cdot H_{\max}^{2}}$$

Where f and H_max_ are the frequency and peak-magnetizing field respectively.

### 3.3 Simulation of local heating effects of single UCP near air-water interface

**Figure 2:**
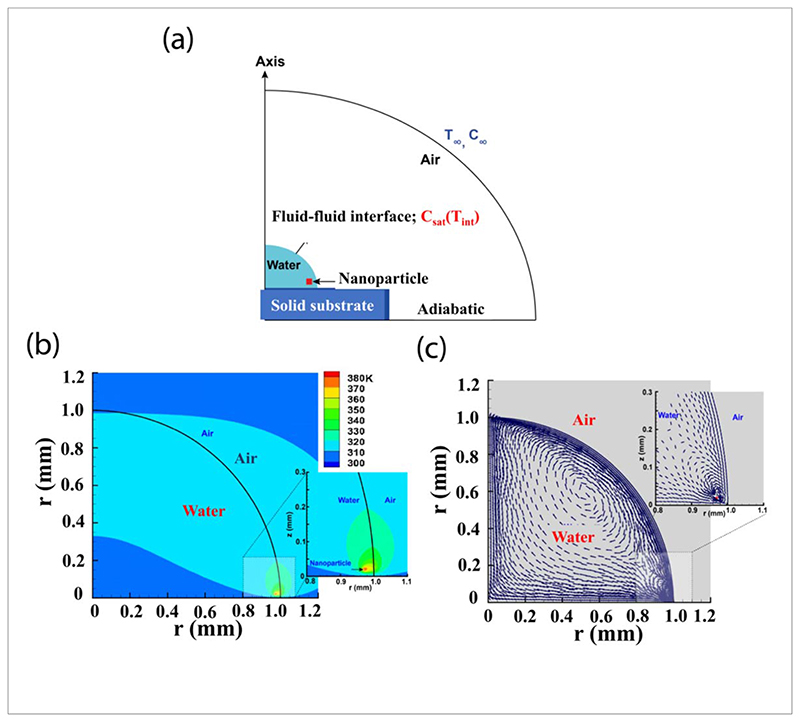
(a) The schematic representation of the numerical model alongwith boundary conditions is shown. (b) Shows the temperature distribution in the vicinity of the particle. (c) shows the velocity field plots of convection currents

The numerical simulation is carried out using the commercial software COMSOL Multiphysics 5.6. A schematic representation of the physical model along with the boundary conditions is summarized in figure 2(a). A sessile drop of water (radius = 1 mm) is placed on the solid substrate. It is assumed that a constant static contact angle (90°)C between the water droplet and the substrate is maintained during the whole simulation period. The nanoparticle (3 *μ*m × 3 *μ*m) on which the Laser beam is targeted is placed near (r = 30 *μ*m, z = 20 *μ*m) the three-phase contact line. In the present simulation, a two-phase flow is coupled with heat transfer and water vapor transport. The governing equations for mass, momentum, energy and the water vapor transport considered for the present problem are discussed below.

#### Mass conservation

(14) $$\frac{\partial \rho }{\partial t}+\nabla \cdot (\rho \text{u})=0$$

*Momentum conservation*:

(15) $$\rho \frac{\partial \text{u}}{\partial t}+\rho (\text{u}\cdot \nabla )\text{u}=\nabla \cdot (-p\text{I}+\text{K})+\text{F}$$

Where, the shear stress tensor **K** can be written in the following form,

(16) $$\text{K}=\mu (\nabla \text{u}+{\left(\nabla \text{u}\right)}^{T})-\frac{2}{3}\mu (\nabla \cdot \text{u})\text{I}$$

In the present study, only the effect of surface tension is considered and is added in the momentum equation (the last term in the R.H.S of equation (15)).

#### Energy conservation

(17) $$\rho C_{p}\frac{\partial T}{\partial t}+\rho C_{p}\text{u}\cdot \nabla T+\nabla \cdot \text{q}=Q+Q_{ud}$$

The heat flux term in equation (17) is expressed as,

(18) q=λ∇T

Further, the first term of R.H.S of equation (17) is the heat source term defined at the fluid-fluid (water-air) interface which can be expressed as follows,

(19) $$Q=Q_{b}=-JL_{H}$$

In equation (19), the term J and L_*H*_ represent evaporative mass flux and latent heat of evaporation respectively. Evaporative mass flux at the interface is evaluated from the water vapor diffusion as,

(20) $$J=M_{w}\text{n}\cdot (-D\nabla c)$$

Where, M_*w*_, n, and D represent the molecular weight of water vapor, surface normal, and diffusion constant (0.282cm^2^ / s) of water vapor in air respectively. The water vapor concentration (c) is evaluated by solving the following species transport equation,

(21) $$\frac{\partial c}{\partial t}=\nabla \cdot \text{J}+\text{u}\cdot \nabla c=0$$

The second term of the R.H.S of equation (17) represents the viscous dissipation.

#### Interface conditions

At the water-air interface, the following conditions are ensured along with the heat source term as defined previously in equation (19),

(22) $$n_{1}\cdot (T_{1}-T_{2})=\sigma (\nabla_{t}\cdot n_{1})n_{1}-\nabla_{t}\sigma$$

Where, *σ* is the surface tension coefficient (0.07 N/m) and T is the total stress which can be expressed as **T** = −p**I** + **K**.

(23) $$u_{1}=u_{2}+M_{f}(\frac{1}{\rho_{1}}-\frac{1}{\rho_{2}})n_{1}$$

At the interface, saturation vapor pressure and saturation vapor concentration are calculated using the Antoine equation as follows,

(24) $$\log({𝒫}_{\text{sat}})=A-\frac{B}{C+T}$$

Where, A, B, and C are the constants for the Antoine equation and T is the temperature.

(25) $$c_{sat}=\frac{{𝒫}_{\text{sat}}}{R_{s}\;TM_{\text{w}}}$$

Here, R_*s*_ is the specific gas constant.

The well-known PARDISO solver is used to solve the coupled equations for the present problem. The BDF method is adopted for the time stepping and the constant (Newton) is selected for the nonlinear method.

The temperature distribution as well as velocity field distribution, are shown in figures 2(b) and (c) respectively. Initially, the temperature of both the water and air zone is fixed at 293.15 K. To replicate the experimental condition, the wall temperature of the nano-particle is fixed at 380.15 K.

Observation reveals that as the hot nano-particle is placed near the three-phase contact line, it results in an enhancement of the evaporative flux across the air-water interface. This is also attributed to the velocity field distribution (figure 2(c)). The convection strength (velocity magnitude) is found to be higher near the interface. However, it reduces near the nano-particle zone in comparison to the zone near the interface. As a consequence, the maximum temperature is observed (about 378K) in the vicinity of the particle position. It should be noted that we have not simulated the phase change process here. During the experiments, the bubble formation occurs near the hot spot shown in the simulation. It is found (figure 2(b)) that a temperature gradient is established (zone of influence) only near the nano-particle while the rest of the domain remains almost undisturbed.

## 4 Results and discussions

### 4.1 Photothermal and magnetothermal effects in a bulk sample

**Figure 3:**
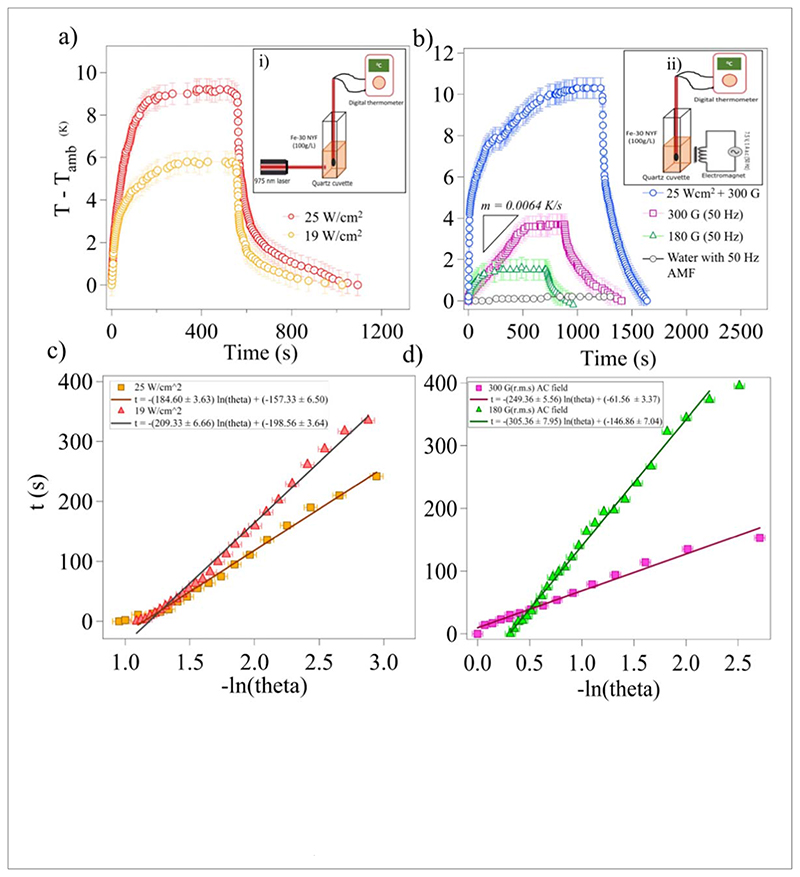
(a) The measured temperature change over 20 minutes with Fe-30 NYF (100g/L) after 975 nm Laser irradiation of different power densities till the temperature saturates, following the Laser being turned off. (b) The Measured temperature change of Fe-30 NYF (100g/L) when exposed to an AC magnetic field of two different flux densities and frequency of 50 Hz. The field is turned off once the temperature of the sample is saturated. The blue curve indicates the combined heating effects of the Laser and magnetic field. A total increase of 10.3 °C is observed. The schematics of the experiments are shown in insets (i) and (ii). (b) and (c) represent the linearly fitted time v/s ln(*θ*) graph of the cooling period in optical and magnetic heating experiments respectively, which is used for the calculation of the thermal conversion efficiency of Fe-30 NYF. The time constant (*τ*) for heat transfer of the system is found to be, *τ_Laser_* = 184.6s at 25Wcm^−3^ power density and *τ_magnet_* = 249.38s for B = 300G(p-p) at 50 Hz.

The photothermal effects of Fe-30 NYF particles are evaluated using a 975 nm Laser at different power densities. The emission spectrum of a3 single Fe-30 NYF by illuminating it with a 975 nm Laser is shown in figure 1(b). For a bulk sample, under the illumination of a Laser at a power density of 25Wcm^−2^ for 10 min, we observe a change of 9.1 °C in the apparent temperature of the sample containing 100 mg of Fe-30 NYF in 1 ml of water which is almost three times higher as compared to pristine NYF particles, where it showed a rise of 2–3 °C with similar power density values[39]. It has been reported that the photothermal conversion properties of pristine NYF particles can be enhanced through La^3+^ doping [39]. However, under almost similar conditions, the particles we report perform better in terms of photothermal conversion. The temperature change of 1 ml of pure water under the same conditions is measured to be 0.3°C. Once the temperature is saturated, we turn off the Laser radiation, and the apparent temperature profile of the Fe-30 NYF sample is observed to follow the cooling curve of water to reach the ambient temperature as shown in figure 3(a). Hence, we confirm that the temperature change observed in Fe-30 NYF sample compared to water could be attributed to its absorption of NIR radiations (975 nm), cause rapid excitation of electrons followed by non-radiative relaxations to lower energy states, leading to the generation of large amounts of heat energy. According to the methods which are already in the literature, the law of conservation of energy can be exploited to find the photothermal conversion efficiencies of the system [33, 40, 41]. We report a photothermal conversion efficiency of 19.4% for a 100mgmL^−1^ of Fe-30 NYF sample under the irradiation of 975 nm Laser with a power density 25 W cm^−2^. For a lesser power (19 W cm^−2^), the observed change in the apparent temperature of the same sample is 5.6 °C.

Further, the magnetic hyperthermia method used to generate heat from superparamagnetic or ferromagnetic microparticles through Neel or Brownian relaxation is well known [24, 25, 42]. It uses high-frequency magnetic fields (in the order hundreds of kHz) to heat up the particles. Since Fe-NYF particles we report have an intrinsic ferromagnetic behavior, it is possible to exploit the thermal energy generated from their hysteresis cycles to heat up their vicinity. The net heat lost per cycle per kg is found from the VSM curve of Fe-30 NYF sample (figure 3(b)). It is then converted into joules by multiplying the mass density of the sample. It is observed that Fe-30 NYF particles reach a saturation magnetization of 3.5 Am^2^/kg. For 100mg/mL of Fe-30 NYF sample, the rate of loss of energy due to the hysteresis is found to be 539.3 mW when we apply an the alternating magnetic field of flux density 300G(peak to peak) at a frequency of 50 Hz. Detailed calculations are followed. The temperature of pure water does not change with the same field. Hence, it is possible to ascertain that the apparent temperature change we measure is caused only by the hysteresis losses of the sample. The temperature of the system is saturated after 10 minutes and an increase of 3.9 °C is recorded as shown in figure 3(b). At this magnetic flux density, the thermal conversion efficiency of Fe-30 NYF particle is determined to be 9.02%. By combining the effects of optical heating and magnetic heating simultaneously, it can be seen that the temperature of the same sample rises by 10.3 °C above the ambient temperature.

The photothermal and magnetothermal conversion efficiencies quantify the degree of heating of the particle and the heat transfer to the medium. From section 3.1, The graph between t and ln(*θ*) for two different Laser powers is shown in figure 3(c). From which, the time constant of heat transfer, *τ* is found to be 184.6 s (corresponding to a Laser with a power density of 25 W cm^−2^. And from equation (5), hS = 18.9 mW K^−1^. Also A_975_ = 0.902, I = 25 W cm^−2^ and T_max_-T_*amb*_ = 9.1 °C, then from equation (6), *η* is found to be 19.42% at 25Wcm^−2^.

Further, the magnetothermal conversion efficiency can also be calculated from section 3.1. Here, the energy lost per cycle per second (*W/Kg*) is given by the area under the hysteresis curve which is shown in figure 1 (c). The area under the VSM curve using numerical integration is, *A* = 35 mJ. The mass density of the particles is found to be 5452.87 kg m^−3^ from diffusive reflection spectroscopy (DRS). Net heat lost by the system during our experiment is given by, *H_tot_* × (*t_T_*=*T*_max_/*t_vsm_*). Hence, the rate of loss of energy by the particles is given by, *Q_h_* = 549.30 mW. And the value of *hS* is given from the time constant as *hS* = 16.84 mW/K. Then, the thermal conversion efficiency from equation (11) yields the value, *γ* = 9.02%, for a peak-to-peak AC field of 300 G.

From section 3.2, the specific loss power and intrinsic loss power are calculated. In our experiments, *C_v_* = 4186.2 J/kg·K (for water), X_*Fe-NYF*_ = 0.1 and *dT/dt* = 0.0064 K/s (obtained from the slope of 300 Gcurve in figure 3(b)). The SLP of the system is then found to be 267.8 W/kg. From the same section, ILP can also be determined. Here, we set f = 50 Hz and H_max_=2.3810^4^ A/m^2^. Hence, ILP is calculated to be 9.46 nH·m^2^/kg.

This is the first time that magnetically induced heating has been shown with upconversion particles. We also show that by combining photothermal and magnetothermal effects, it is possible to increase the temperature of the same sample by 10.3 °C as depicted in figure 3(b). In this manner, we are able to increase the maximum temperature of the sample by another 1.2 ° C from photothermal saturation.

### 4.2 Heat generation with single Fe-NYF particle

**Figure 4:**
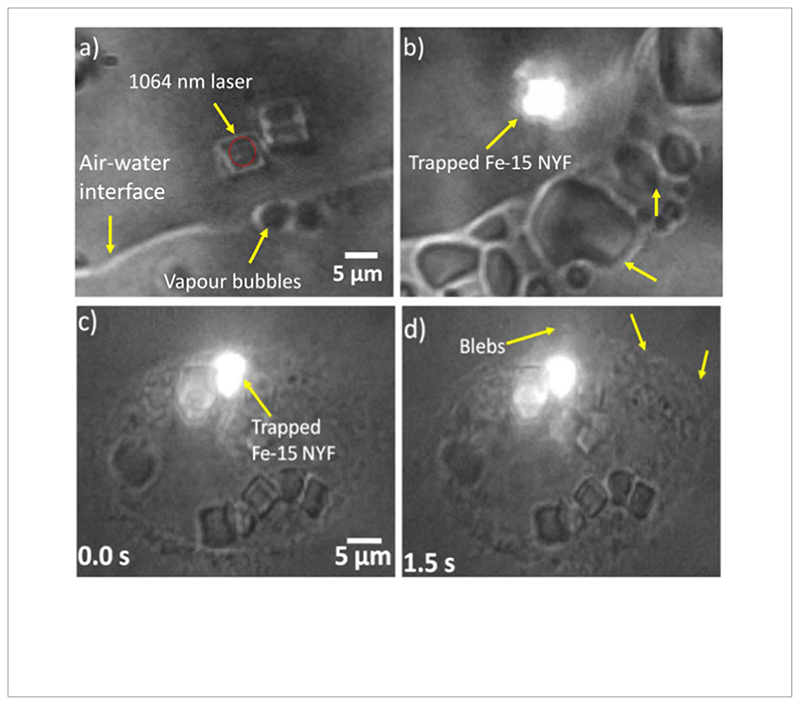
The initiation of boiling of water to form vapor bubbles, at the air-water interface due to the heating of a Fe-15 NYF particle, illuminated with (a) 1064 nm Laser at 55 mW power and (b) 975 nm Laser (where the visible emission from the particle appears as a white spot on the image, with spectrum in figure 1) at 11.4 mW power are shown. In figures (c)–(d), the blebbing of MCF-7 cells caused by the heating of an optically trapped Fe-15 NYF upconversion particle inside the cell is shown. The particle is trapped with 975 nm Laser at a power of 22 mW.

We show that the particle is optically trappable and the power spectrum of the trapped particle is shown in figure 1(c). Then, we direct our attention to measuring the thermal absorption properties of the particle. For this, we take one such particle and place it close to the side interface of a sessile water droplet on the glass cover slip. The particle is then illuminated with 1064nm light and 975 nm light sequentially. We look for the Laser power at which it starts to form bubbles on the side interface. We find that the threshold for bubbling on the side interface for 1064nm light is 55 mWat the sample plane, while the same threshold power at 975 nm is 11.4 mW. Assuming that the formation of the bubbles are a consequence of the local environment of the droplet reaching the boiling point of water, we find that the Fe-NYF is 5 times less absorptive at 1064nm than at 975 nm. This effect has been shown in figures 4(a) and (b) respectively. To ascertain that the particle is responsible for the absorption of the light and subsequent heating, we illuminate the 975 nm Laser beam without the particle and find no bubbles at the surface. Thus, the particle is indeed responsible for absorption and subsequent heating. It may be noted here that water itself is about 3 times more absorptive at 975 nm than 1064 nm[43], which might partially account for an increase in heating efficiency. It may also be noted here that the figure 4(b) shows a white patch which is the visible emission from the particle upon being illuminated by the 975nm light with a spectrum shown in figure 1. The backscattered infra-red light does not appear in the camera due to the dichroic in the backscatter direction which filters out any wavelength higher than 750 nm while only allowing visible light to pass.

The heating effects of an optically trapped Fe-NYF on its local environment are simulated near the air-water interface using commercially available COMSOL software. The configuration of the simulation is shown in figure 2(a). We find that a hot nanoparticle placed near the triple contact line in a sessile water droplet heats up its immediate surroundings and increases the local evaporative flux at the boundary, thus the convection flow strength (See figure 2(b)). As a consequence, we observe the formation of vapor bubbles which indicates the particle-induced, rapid evaporation of water near the air-water interface as shown in figure 4(b).

We also show a typical application of the heating of the particle by the Lasers. We make cancer cells ingest the particles by phagocytosis and then illuminate them with a Laser. When we illuminate the particle with about 100 mW of 1064nm Laser power, the cells die in seconds. The effect of killing the cell is also seen upon the usage of 975 nm Laser at 22 mW power. We observe the initiation of blebsonthe cell membrane when a Fe-15 NYF is illuminated with 975 nm Laser within the cell for 308 seconds (Mean ± SD, for N=6, at 22 mW power). This is an indication of induced cell death caused due to the enormous absorption of 975 nm radiation by the particle, resulting in the heating of its local environment, as shown in figures 4(c)–(d). If we prefer to not kill the cells, we use about 30 mW of 1064 nm Laser power and trap the particles while keeping the cell alive for more than 5 minutes. This also implies that the particles are biocompatible. This is the highest Laser power applied to a continuous particle having a high magnetic moment with low heating.

Further, local heating could be performed with a combination of optical and magnetic fields. The particle can be brought to the desired location with magnetic fields and then either alternating magnetic fields used or infra-red Laser used to heat up the local neighborhood, particularly if the particle is close to the skin surface.

Although the extinction coefficient of Fe-30 NYF particles we report is less than that of gold (≈0.15[44]) and other metallic particles under 975nm illumination, they show a very efficient photothermal conversion without relying on plasmonic effects [45]. The refractive index of Fe-30 NYF particles has been reported to be 1.478 ± 0.0029 *ι* [21]. In this regard, we also envisage that the photothermal properties of our particles can be improvised further by introducing a core–shell configuration with metallic nano/microparticles. We would also like to point out that unlike gold and the majority of other metallic nanoparticles, our particles exhibit residual ferromagnetism that allows low-field, low-frequency magnetic heating them. Whereas gold nanoparticles are either paramagnetic or mild, soft-ferromagnetic [46]. While coming to *in-vivo* applications, Fe-30 NYF particles can be used as illumination probes to provide better imaging and as photo/magnetothermal probes simultaneously, which makes these particles superior to gold and other metallic nanoparticles.

## 5 Conclusion

In conclusion, we have shown Fe-30 NYF is more efficient than NYF towards the photothermal effect. It also exhibits low-frequency magnetically induced heating. We have also calculated the photothermal and magnetothermal efficiencies for bulk Fe-NYF. We envisage a combination of optical and magnetic fields to attain high heating efficiency. Further, these particles can possibly be inserted even inside bloodstream to direct them towards diseased regions of the body magnetically and then induce cell death towards cancer therapy. The exact modalities of such therapy in-vivo are a matter of future work. It is also known that infra-red light penetrates the skin more than visible radiation [47–49]. Thus, one may also envisage tracking the particles flowing through the blood vessels close to the surface while moving them magnetically through the blood.

## Data Availability

This is a material and all information about making the material is available in the manuscript. The data that support the findings of this study are available upon reasonable request from the authors.
